# Investigating the Feasibility, Acceptability, and Appropriation of a Socially Assistive Robot Among Minority Youth at Risk of Self-Harm: Results of 2 Mixed Methods Pilot Studies

**DOI:** 10.2196/52336

**Published:** 2023-11-22

**Authors:** A Jess Williams, Ellen Townsend, Nkem Naeche, Amelia Chapman-Nisar, Chris Hollis, Petr Slovak, Digital Youth With Sprouting Minds

**Affiliations:** 1 Department of Informatics King's College London London United Kingdom; 2 Institute of Mental Health University of Nottingham Nottingham United Kingdom; 3 See Acknowledgments

**Keywords:** digital intervention, self-harm, young people, emotion regulation, experience sampling, interviews

## Abstract

**Background:**

Minority youth are at an increased risk of experiencing self-harmful thoughts and behaviors. However, there is limited evidence of successful interventions to support young people in the moment of their distress. Digital interventions are considered a potential solution for providing in-the-moment support for those at risk of adverse mental health and self-harm.

**Objective:**

These pilot studies aim to investigate the feasibility and acceptability of a new in situ intervention tool, Purrble, among two broad groups of minority youth: (1) lesbian, gay, bisexual, transgender, queer, and similar minority (LGBTQ+) youth and (2) racial and ethnic minority youth. Purrble was designed to support in-situ emotion regulation (ER) support when individuals are facing emotionally challenging situations.

**Methods:**

This study consisted of 2 mixed methods pilot studies that followed the same mixed methods design, including 3 weeks of daily and weekly surveys and optional follow-up interviews. Inclusion criteria were (1) aged between 16 and 25 years, (2) part of a minority group, (3) had experiences of self-harmful thoughts or behaviors or elevated symptoms of depression or anxiety, and (4) living in the United Kingdom at the time of the study. The primary outcomes were (1) the feasibility of Purrble as an intervention among pilot samples (analyzed by consent rate, retention rate, adherence to surveys, and engagement with the device) and (2) the acceptability and appropriation of Purrble across pilot studies as a tool to support ER in situ (thematically analyzed qualitative open-ended questions and interview data). The secondary outcomes were descriptive pilot data concerning the mental health outcomes in each sample.

**Results:**

In total, 21 LGBTQ+ young people participated in pilot study 1, with 86% (n=18) completing the baseline and 3 weeks of daily surveys. These young people maintained engagement with Purrble across deployment, across which period there was a decrease in self-harmful thoughts and anxiety symptoms. A total of 19 ethnic and racial minority youths participated in pilot study 2, and 84% (n=16) completed the study. Although pilot study 2 participants also maintained engagement with Purrble across deployment, this was to a lesser degree than participants of pilot study 1, and perceived mental health outcomes did not indicate potential change associated with the device. The thematic analysis indicated three superordinate themes: (1) stopping the self-harm cycle, (2) adopting ER strategies, and (3) stages of change.

**Conclusions:**

These were the first pilot studies of a novel intervention that aimed to provide in situ ER support for young people at risk of self-harm. Both quantitative and qualitative findings indicate that young people found Purrble to be a feasible and acceptable intervention, as they effectively incorporated the device into their ER practices. These engagements with Purrble were described as interrupting the cycle of self-harmful ideation and behavior.

## Introduction

### Background

Self-harm, the injury or poisoning of self irrespective of suicidal intent [1], is a prominent and increasing concern among young people [2-5]. Between the ages of 16 and 25 years, around 15.7% of young people engage with self-harm [5], with high risk of repetition [6,7] and escalation [8]. Given that previous self-harm is the strongest risk factor associated with completed suicide [9], supporting those at risk of, and engaging with, self-harm is a key aspect of suicide prevention.

Prominent risk factors for self-harm among youth populations include mental health difficulties (eg, anxiety and depression), interpersonal difficulties (eg, bullying), and sociodemographic factors such as being part of a minority group [6]. It has been well documented that being part of a minority group is associated with experiences of stigma, prejudice, and discrimination [10-13]. Those at risk of such experiences are recognized as experiencing poorer interpersonal relationships [14], worse mental health outcomes [12], and an increased risk of self-harm and suicide [2,13]. Recent research with young people has highlighted two minority populations that are likely to be at greater risk of self-harm: (1) those who identify as lesbian, gay, bisexual, transgender, queer, and similar minority (LGBTQ+) [2] and (2) ethnic and racial minorities [15,16].

In brief, the prevalence of self-harm is notably higher among LGBTQ+ young people than among cisgender, heterosexual peers [17-19]. Within the United Kingdom alone, 65.3% of LGBTQ+ youth have self-harmed, with 25.7% going on to make a suicide attempt [20]. Although rates have remained high among White populations, increased rates of young people from racial and ethnic minority groups have been presenting to hospitals for self-harm in the past decade [16]. Furthermore, these young people were more likely to live in high deprivation and report difficulties within their families [16], these factors independently contribute to the risk of self-harm which are both independently risks for self-harm [21-23].

Those who struggle with self-harm and suicide experience difficulties with emotion regulation (ER) [24-26]. ER is widely defined as the implicit or explicit attempt to recognize, understand, and manage emotions [27-29]. In a recent meta-analysis of 48 publications, a significant association (odds ratio 2.40) was found between emotion dysregulation and self-harm [24], with evidence indicating that this was related to a lack of emotional awareness and inability to access alternative ER strategies. Qualitatively, this has been supported by young people recognizing that poor ER abilities and strategies, such as suppression, lead to worsened mental health, specifically anxiety attacks and self-harm [30].

Few psychological interventions focus on self-harm among young people [31-33]. A recent Cochrane Review found no clear supportive evidence for cognitive behavior therapy, mentalization-based therapy, group-based psychotherapy, family interventions, or remote contact interventions in 17 randomized control trials [31], mirroring previous findings that suggested that cognitive behavioral therapy evidence was variable [32]. Although there was some effectiveness of dialectical behavior therapy among adolescents (odds ratio 0.46), this was a relatively small effect size [31]. Furthermore, such intensive interventions often rely on delivery mechanisms, such as workshops or in-person sessions, which do not support the application of intervention skills in situ [34]. This research highlights the lack of appropriate treatment to support and prevent young people who are at risk of self-harm.

Therefore, this study aims to evaluate a new potential intervention, Purrble, a socially assistive robot designed to support in-the-moment ER in daily life [35,36]. Across children and student populations, Purrble has been well accepted and has been associated with notable benefits, such as supporting ER strategies and reducing anxiety [35-38]. However, to date, there has been no research to understand how young people at risk of self-harm may engage with Purrble.

### Objectives

This study aims to determine whether Purrble is a feasible and acceptable intervention among young people at a high risk of self-harm. The specific objectives are as follows:

- To determine the feasibility of Purrble as an ER intervention by considering retention and engagement rates.
- To consider the acceptability and appropriation of Purrble by exploring young people’s thoughts and experiences of Purrble.
- To understand young people’s experiences of using Purrble in relation to their emotional distress and self-harm.

## Methods

### Overview

**Figure 1:**
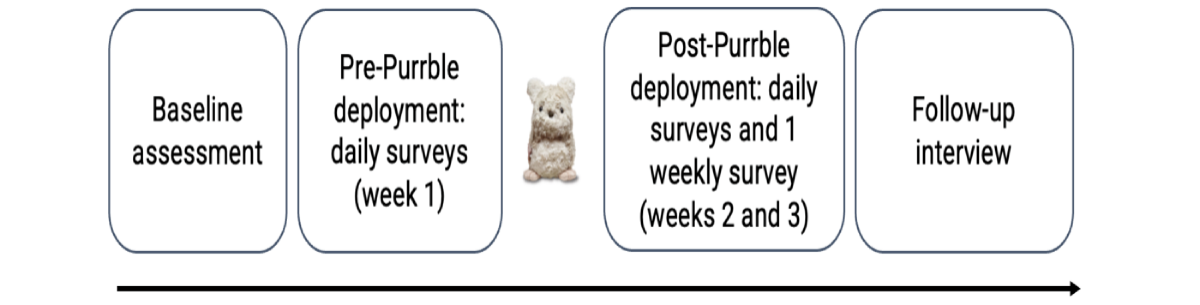
Data collection phases for both pilots.

This study consisted of 2 independent pilot studies examining the impact of access to Purrble for two subgroups of minority youth at risk of self-harm: (1) LGBTQ+ youth and (2) racial and ethnic minority groups. These pilot studies followed the same mixed methods design using daily and weekly self-report surveys, with optional follow-up interviews, as shown in Figure 1. The design was discussed with members of the UK Research and Innovation–funded Digital Youth Research Programme advisory group, Sprouting Minds. Throughout the process, these Sprouting Minds members were consulted on various study elements, such as recruitment procedures, how to engage with different minority groups, and dissemination.

### Participants

#### Pilot Study 1

LGBTQ+ participants were recruited between May and July 2022 using social media platforms and MQ’s research website Participate. To take part, participants had to meet the following criteria: (1) identify as any part of the LGBTQ+ umbrella, (2) have experiences of self-harmful thoughts or behaviors in the past 6 months or have moderate symptoms of anxiety (Generalized Anxiety Disorder-7; GAD-7) or depression (Patient Health Questionnaire-9; PHQ-9), (3) be aged between 16 and 25 years, (4) currently be living in the United Kingdom, and (5) have access to a mobile phone.

#### Pilot Study 2

Black and ethnic minority participants were recruited using the same strategies mentioned earlier. However, owing to low engagement, additional advertising was provided by McPin as recommended by a Sprouting Mind member. Recruitment was conducted between September and November 2022. The inclusion criteria were the same as in study 1, except that young people had to identify as part of an ethnic or racial minority, rather than identifying as LGBTQ+.

Symptoms of anxiety and depression were selected as inclusion criteria for each pilot sample, as these are known indicators of risk for self-harm in adolescent and young populations [6]. In addition, adverse mental health outcomes among minority groups are known to increase the risk of self-harm or suicide [12,18,19].

### Intervention

**Figure 2:**
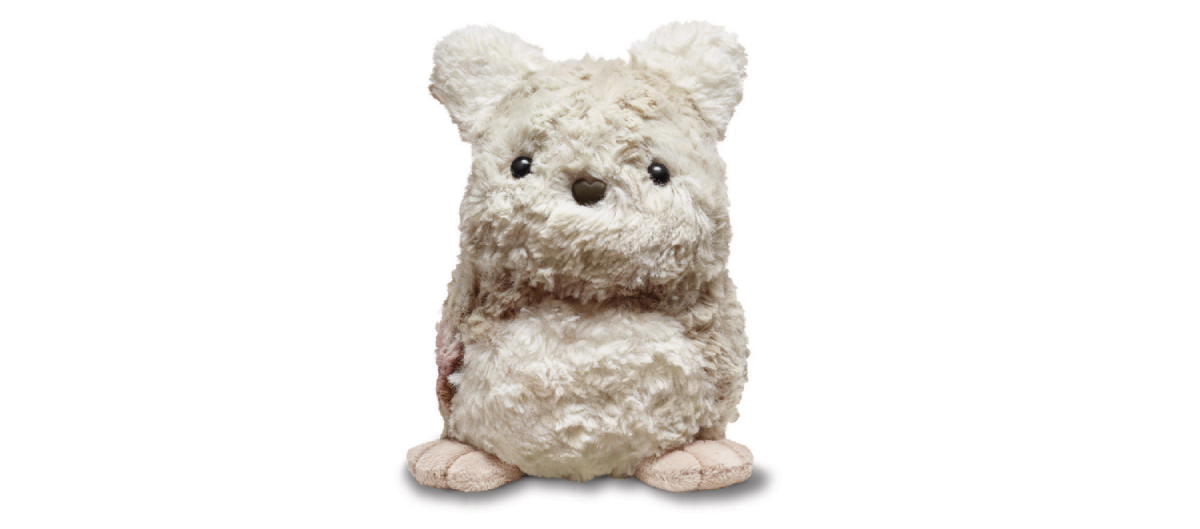
Purrble intervention device.

The intervention takes the form of an interactive plush toy (Figure 2), known as Purrble, which aims to guide the user to downregulate their unwanted emotions through a combination of sensors and haptic vibrations, with the theory of change targeting the attentional deployment and response modulation components of the ER process [35,36]. The smart toy’s internal state is communicated to users through vibration patterns that mimic a heartbeat, with faster rates corresponding to higher “stress” levels. The fundamental game loop is as follows. Whenever the smart toy wakes from sleep, it is startled and has a rapid heartbeat. Shakes, sudden movements, or pressing its ears also wake and “startle” the toy, whereas calm stroking movements and hugs gradually slow the heartbeat, which eventually changes to a purring vibration, indicating a calm, happy state. The toy also produces varying gentle sounds (ie, sighs, coos, giggles, and grunts) that correspond to its internal state and complement the vibration-based feedback. Full details, including the association with the theory of change model, are available in previous research [35,39].

### Ethical Considerations

Ethics approval was obtained from King’s College London (HR.DP-21.22-28826) before the start of the investigation. Interested individuals were able to download an information sheet outlining the study, inclusion criteria, their rights as participants, and how to contact the researchers or ethical committee for any questions, alongside a web-based consent form via King’s College London Qualtrics. Individuals were asked to confirm their understanding of the study, that they wished to voluntarily take part in the study, that they understood their rights as participants, how data would be anonymized and handled, and that they were happy to share their contact information and sign the bottom of the form. These forms explained that all data would be made anonymous upon completion of the study, with ID numbers being used for data linkage.

Additional consent was obtained from those who wished to participate in follow-up interviews. The consent forms were sent by email to the participants within each pilot study in the final week. The consent forms outlined the requirements of the study (audio-recording) and explained the process and how data would be anonymized and handled.

As compensation, all participants kept their Purrble devices (equivalent to approximately £55 [US $66.52]) and were offered an additional £10 (US $12.11) for their time to participate in the interview.

### Procedure

The study design for both pilot studies included several data collection phases (Figure 1): (1) baseline assessment, (2) experience sampling over a 21-day period (1 daily survey at 6 PM, with a reminder at 7 PM), (3) follow-up interviews, and (4) co-design workshops. In this study, we report the first 3 phases.

All assessments were conducted on the web using Qualtrics (Silver Lake). The baseline assessment was advertised through various recruitment streams, containing a downloadable information sheet and a digital consent form. This was used to ensure participants’ inclusion by considering responses to demographic questions (eg, age, country of residence, sexual orientation, gender identity, ethnicity, and age) alongside measures of anxiety; depression; emotional regulation; and whether someone had experienced self-harmful thoughts, suicidal thoughts, or self-harmful behaviors in the last 6 months.

Once the baseline assessment was reviewed, participants were invited to a study briefing via Zoom (Zoom Video Communications) at their own convenience. During this briefing, the study was explained in detail, participants were reminded of their rights, initial safeguarding was conducted (Multimedia Appendix 1 [40,41]), and participants’ contact information (eg, phone number and address) was checked. Participants were also told that once they received Purrble, it was theirs to keep, and it was not expected to be returned at the end of the study or in the event that they withdrew. At all points, the participants were invited to ask questions.

The 21-day experience sampling measures (ESMs) period ran from the next consecutive week following participant briefing. Daily ESM surveys were administered via text message as an embedded Qualtrics link, which was sent at 6 PM each night, with a reminder text for those who had not completed the survey at 7 PM.

During week 1, the ESM survey mirrored the baseline assessment using ESM-adapted items, asking about mood [42], ER [43,44], and thoughts of self-harm [43]. During this week, Purrble was sent to the participant’s nominated address with instructions to open on the Monday of week 2. From this date, participants’ ESM surveys an additional item that queried engagement with Purrble during that day [37]. Daily surveys took 2 to 3 minutes across the 21-day ESM period.

Participants were also sent a secondary questionnaire (approximately 2 min) via text message on the Sunday of weeks 2 and 3, at 7.30 PM, with a reminder an hour later. This asked the participants more broadly about their engagement with Purrble and their perception of the technology [42].

Following week 3 of the ESM period, participants were sent an email thanking them for taking part in the study and inviting them to a semistructured interview (interview topic guide: Multimedia Appendix 2). This was highlighted as optional, and those who did not wish to take part or did not respond were sent a debrief sheet. If participants wished to participate, an interview was arranged at the participants’ convenience following the receipt of a completed, signed consent form. Interviews took place over Zoom and were audio recorded. The participants were encouraged to speak freely about their opinions, perceptions, and experiences of Purrble. The interviews lasted an average of 33 (SD 9.3; range 17-50) minutes.

### Measures

#### Phase 1: Baseline Assessment

##### GAD-7 Measure

GAD-7 is a 7-item scale used to screen for the presence and severity of general anxiety using a 4-point Likert scale ranging from 0 (not at all) to 3 (nearly every day) [45]. Scores of ≥10 indicate moderate levels of anxiety. Across the 2 pilot studies, Cronbach α ranged between .77 and .94.

##### PHQ-9 Measure

PHQ-9 is a 9-item scale used to score the severity of depression on a 4-point Likert scale [46]. Higher scores indicate more severe clinical symptoms of depression, with scores ≥10 indicating moderate levels of depression. Cronbach α scores ranged from .80 to .92 between the 2 pilot studies.

##### Difficulties in ER Scale-18

The Difficulties in Emotion Regulation Scale-18 is an 18-item questionnaire that assesses how well participants feel they regulate their emotions using a 5-point Likert scale ranging from 1 (almost never) to 5 (almost always) [47]. The Difficulties in Emotion Regulation Scale-18 includes 6 dimensions of emotional regulation: emotional awareness, emotional clarity, emotional acceptance, effective use of ER strategies, goal-related behavior, and impulsivity during negative mood. Cronbach α was .86 for pilot study 1 and .91 for pilot study 2.

##### Beliefs About Emotion Regulation (ER Beliefs)

This 4-item measure was adapted from the Implicit Beliefs about Emotions scale [48,49]. This assesses personal beliefs about the malleability of emotions by considering incremental and entity beliefs. A 5-point Likert scale is used, ranging from 1 (strongly disagree) to 5 (strongly agree). In these samples, internal consistency was 0.71-0.83.

##### Self-Harm

Participants were asked 3 binary questions regarding their self-harmful experiences in the past 6 months. These questions asked whether they had experienced self-harmful thoughts, suicidal thoughts, or self-harmful behavior.

#### Phase 2: 21 ESM Items

The ESM items (see overview in Table 1) were selected to mirror the constructs measured at baseline. All items used were obtained from ESM Item Repository [50] from 2 primary sources: SUPREME CORT (adapted GAD-7 and PHQ-9 [42]) and the SIGMA study [51]. Additional items were sought from the repository to relate to emotional regulation [44]. All items were presented as consistent visual analogs using Likert scales, which have been suggested to reduce participant burden [52]. For more information, refer to Multimedia Appendix 3.

The weekly survey contained only 1 measure, the Twente Engagement with E-health Technologies Scale (TWEETS) [53]. This assessed the digital engagement of Purrble across 9-items using a 5-point Likert scale, ranging from strongly disagree (0) to strongly agree (4). Across pilot study 1, the Cronbach α was .74, whereas it was .79 in pilot study 2.

**Table 1:** Overview of daily experience sampling measures (ESMs).

Origin of item	ESM measure	Items, n	Scoring
SUPREME CORT [42]	Anxiety symptoms (GAD-7^a^)	7	0 (not at all) to 3 (nearly every day)
SUPREME CORT [42]	Depressive symptoms (PHQ-9^b^)	9	0 (not at all) to 3 (nearly every day)
[43,44]^c^	Emotion regulation	4	1 (not at all) to 7 (a lot)
SIGMA [51]	Self-harm thoughts	1	1 (not at all) to 7 (very much)
[37]^c^	Engagement with Purrble	4	Varying depending on item wording

^a^GAD-7: Generalized Anxiety Disorder-7.

^b^PHQ-9: Patient Health Questionnaire-9.

^c^These studies are not cited from a named project of work.

### Analysis

Statistical data were analyzed using RStudio (Posit PBC). For baseline measures, a descriptive analysis was performed to provide insights into the characteristics of the pilot samples. The feasibility of the intervention was assessed in four ways using descriptive statistics: (1) consent rate to the study, indicating interest in the intervention; (2) retention of participants across the study period; (3) adherence to daily surveys; and (4) self-reported engagement with the intervention (TWEETS). In addition, weekly averages of total scores (GAD-7, PHQ-9, and ER strategies) and risk ratio of any thoughts of self-harm across the week were calculated to indicate the perceived impact of Purrble on mental health.

Qualitative data from both pilot studies were analyzed using thematic analysis to explore acceptability, participants’ perceptions of Purrble and how this may have related to their self-harmful experiences, and appropriation of Purrble. The thematic analysis followed the 6 phases outlined by Braun and Clarke [54-56] using participant interviews as the primary data source. Following transcription, data were imported into NVivo 12 (Lumivero), and AJW deductively coded the data using line-by-line coding and reflecting on their own practice. Codes were clustered into meaningful preliminary themes, which were discussed with PS. These themes were then triangulated using open-text responses from the daily surveys and data from the co-design workshops. Following this, additional considerations and reflections were discussed, resulting in the revised thematic framework that was reviewed by all the authors. A full analysis of the workshops will be reported elsewhere as part of an ongoing iterative co-design process.

## Results

### Final Samples

Pilot study 1 consisted of 21 LGBTQ+ young people with an average age of 21.4 (SD 2.7; range 16-25) years. Most participants identified as cisgender female (14/21, 67%), with other participants self-labeling as agender, nonbinary, questioning, demiboy, and transgender male. Participants reported a range of sexual orientations, mostly describing themselves as bisexual, lesbian, or queer. All participants lived in either England or Scotland, and most considered themselves White British (17/21, 81%).

Pilot study 2 included 19 participants with an average age of 21.1 (SD 2.8; range 17-25) years. The sample was mainly cisgender females, including 1 cisgender male and 2 nonbinary participants. Much of the sample had individual racial or ethnic backgrounds, 5 participants identified themselves as Asian Chinese, and 2 participants were Pakistani. All participants lived in England and over half were heterosexual (10/19, 53%). Further demographics of both samples are presented in Multimedia Appendix 4.

At baseline, LGBTQ+ youth indicated higher average levels of risk for anxiety and depressive symptoms, as well as more self-harmful thoughts and behaviors in the past 6 months, than ethnic and racial minority young people. LGBTQ+ participants also had slightly higher rates of emotion dysregulation and held relatively fixed beliefs about ER; these findings were similar to those reported by ethnic and racial youth (Table 2).

**Table 2:** Baseline descriptives for both the pilot studies 1 (N=21) and 2 (N=19).

	Study 1: LGBTQ+^a^	Study 2: ethic and racial minorities
Anxiety symptoms (GAD-7^b^), mean (SD)	13.8 (4.0)	12.7 (5.6)
Depressive symptoms (PHQ-9^c^), mean (SD)	13.4 (5.8)	13.2 (7.5)
Self-harmful thoughts, n (%)	20 (95)	16 (84)
Suicidal thoughts, n (%)	13 (62)	11 (58)
Self-harm behavior, n (%)	17 (81)	10 (53)
Emotion dysregulation (DERS-18^d^), mean (SD)	58.2 (12.7)	54.9 (14.5)
Emotion regulation beliefs, mean (SD)	13.2 (3.3)	12.8 (2.0)

^a^LGBTQ+: lesbian, gay, bisexual, transgender, queer or questioning minority.

^b^GAD-7: Generalized Anxiety Disorder-7.

^c^PHQ-9: Patient Health Questionnaire-9.

^d^DERS-18: Difficulties in Emotion Regulation Scale-18.

### Feasibility

Between the 2 pilot studies, a greater number of people expressed their interest in participating in pilot study 1 (N=45) compared with pilot study 2 (N=26). However, a similar number of young people were eligible and provided valid consent and contact information for both studies (Table 3).

Of the 22 consenting participants in pilot study 1, 1 (5%) withdrew owing to illness before the start of the study. A further 2 participants stopped responding to the daily surveys once they had received their Purrble; therefore, the final study retention rate was 90% (19/21). During the study, 1 participant experienced delivery delays for their Purrble, which impacted the timing of their responses. Nevertheless they continued to participate in the study however, their results were excluded from the final quantitative analysis. Across the remaining 18 participants, the average adherence to daily surveys was 18 (SD 3.0) completed surveys per young person (305/378, 80.7%).

A total of 19 participants were enrolled in pilot study 2; however, 3 people dropped out during the 3-week period—they did not respond to any of the surveys and were therefore excluded from the analysis. This resulted in a retention rate of 84% (16/19). Of the 16 remaining participants, an average of 17 (SD 4.3) surveys were completed per young person (265/336, 78.9%).

Within pilot study 1, participants consistently engaged with Purrble to a high degree; there was no clear differences in TWEETS scores between weeks 2 to 3. Similarly, those in pilot study 2, were also consistent in their use of Purrble; however, this was a lower level of engagement than the LGBTQ+ participants. Given the consistent level of engagement with Purrble and similar adherence and retention rates, it appears that Purrble was a feasible and acceptable intervention device within both minority groups.

Using preliminary data, we also briefly explored the perceived impact of Purrble over the 2-week deployment (Table 4).

Among pilot study 1 participants, average scores of anxiety and depressive symptoms were relatively low across the study; there was a consistent decline in anxiety symptoms and a small decrease in depressive symptoms between pre- and post-Purrble deployment. This was mirrored by a reduction of self-harmful thoughts across the study period. However, engagement with ER strategies also appeared to decrease slightly across the study.

Comparatively, pilot study 2 participants reported a decrease in anxiety symptoms between pre- and post-Purrble deployment; however, depressive symptoms and ER strategies fluctuated without a clear pattern. Although self-harmful thoughts were reported less frequently at the final data point, it was not indicative of an overall pattern of reduction. It is possible that the greater differences in mental health outcomes among pilot study 1 can be attributed to higher levels of Purrble engagement, suggesting that Purrble may be a more effective intervention for LGBTQ+ youth.

**Table 3:** Feasibility outcomes for both the pilot studies 1 (N=21) and 2 (N=19).

	Study 1: LGBTQ+^a^	Study 2: ethic and racial minorities
Study response, n	45	26
Consent, n	22	19
Drop out before the study starts, n	1	0
Drop out during the study, n	2	3
Retention rate (%)	86.4	84.2
Adherence: number of surveys completed, mean (SD)	18 (3.0)	17 (4.3)
Adherence: number of surveys completed, n (%)	305 (80.7)^b^	265 (78.9)^c^
TWEETS^d^ week 2, mean (SD)	28.3 (3.7)	21.2 (6.8)
TWEETS week 3, mean (SD)	28.3 (4.4)	21.6 (5.6)

^a^LGBTQ+: lesbian, gay, bisexual, transgender, queer, and similar minority.

^b^N=378.

^c^N=336.

^d^TWEETS: Twente Engagement with E-health Technologies Scale.

**Table 4:** Perceived impact of Purrble between predeployment and postdeployment.

Measure		Week 1	Week 2	Week 3
**Pilot study 1**				
	GAD-7^a^, mean (SD)	8.87 (4.95)	6.58 (4.53)	5.94 (4.45)
	PHQ-9^b^, mean (SD)	9.47 (6.56)	8.44 (6.18)	8.45 (7.10)
	ER^c^ strategies, mean (SD)	17.74 (2.86)	16.68 (2.06)	16.30 (2.84)
	Self-harm thoughts, risk ratio	0.73	0.61	0.56
**Pilot study 2**				
	GAD-7, mean (SD)	8.20 (4.72)	7.47 (4.62)	7.40 (4.85)
	PHQ-9, mean (SD)	9.36 (6.37)	7.49 (5.25)	9.56 (7.13)
	ER strategies, mean (SD)	19.47 (2.90)	17.91 (2.96)	18.85 (4.03)
	Self-harm thoughts, risk ratio	0.33	0.60	0.33

^a^GAD-7: Generalized Anxiety Disorder-7.

^b^PHQ-9: Patient Health Questionnaire-9.

^c^ER: emotion regulation.

### Acceptability and Appropriation

#### Overview

To determine the acceptability of Purrble, young people were invited to participate in a poststudy semistructured interview. This explored their perceptions of Purrble, considering how it might relate to their emotional distress and potentially self-harm. Across all participants, LGBTQ+ young people disclosed more experiences of self-harm than ethnic and racial minority youth. Therefore, Purrble was discussed more closely with LGBTQ+ young people’s experiences of self-harm, whereas ethnic and racial youth tended to focus on emotional distress broadly, with reference to self-harm. A total of 3 themes were developed, each containing subthemes, as shown in Table 5. The details of the themes and subthemes are detailed below with example quotes.

**Table 5:** Thematic framework of Purrble with young minority people at risk of self-harm.

Theme	Descriptor	Subtheme
Stopping the self-harm cycle	Young people described 2 distinct ways when Purrble impacted their self-harm experiences.	Not reaching the point of self-harm ideation Preventing self-harm behavior
Adopting emotional regulation strategies	Participants were given no instructions about how or when to use Purrble, yet a range of emotional regulation approaches were adopted.	Recognize, step back, and control the impulse Comforting and self-soothing Addressing physical manifestations of distress
Stages of change	Young people were clear about when they felt Purrble would or would not be useful.	Stopping the spiral Breaking the habit

#### Stopping the Self-Harm Cycle

Young people who were currently struggling with self-harmful thoughts and behaviors described 2 distinct ways in which Purrble impacted these experiences. For some, Purrble helped them to not reach the point of self-harmful ideation, whereas for others, it could also be used to prevent people from acting on their self-harm impulses.

##### Not Reaching Self-Harm Ideation

During the study, some young people reported experiencing fewer self-harmful thoughts during the 2-week deployment:

I think I’ve had less thoughts of like self-harming, low mood.

Agwn

This was related to participants using Purrble during times of emotional distress to prevent themselves thinking about self-harm:

...they [Purrble] can help to push them [thoughts] off until I can go and sit in a more public area with my flatmates or whatever or help me distract myself until I can sleep.

Usuv

Such experiences suggested that Purrble was an acceptable intervention to prevent young people from struggling with self-harmful ideation, allowing them to engage in positive coping strategies.

##### Preventing Self-Harm Behavior

More commonly, Purrble was used during moments of distress to prevent self-harm behavior following ideation:

It’s just for me, it’s less so that those types of things would stop me from thinking about it or having a thought about it, but that would be more in terms of that you have the thought and then you don’t act on it.

Fead

Young people described how Purrble drew their attention away from their thoughts of self-harm:

I think when I was feeling more suicidal, it was more like I was focusing on purrble a bit more.

Rhhn

Young people also described how Purrble could be used to distract them from those thoughts, thereby preventing their self-harm:

I mean you can’t self-harm if you’re busy doing something else and also it can just sort of make you forget that you wanted to in the first place. Then you can just end on like 10 tangents doing something else.

Avvm

#### Adopting ER Strategies

##### Overview

Although participants were given no instructions on how or when to use Purrble, they described it using a range of ER approaches. By engaging with Purrble during moments of distress, young people were able to ground themselves, self-soothe, and address the symptoms of their emotions. This suggests that Purrble is an acceptable intervention, as young people were able to effectively use it as an ER tool even without explicit instructions.

##### Recognize, Step Back, and Control the Impulse

Regardless of whether young people engaged with Purrble during points of self-harm or other emotional difficulties, Purrble was used as a way to ground oneself:

...most of the time it was like purrble is in distress and I need to look after purrble and maybe that will help me focus and like ground myself, uhum, I think that’s like one of the main things is to like focusing on the texture, focusing on like stroking this purrble to help you to calm down and just feel more grounded.

Huiq

Young people described that they felt better able to recognize their emotional distress, take a moment to ground themselves, and respond to difficult situations:

This afternoon I really struggled with going into something similar to a dissociative state/flashbacks and I spent a long time going between being aware of reality and shutting down. My husband brought me Purrble to see whether it would help, and I felt that it was a lot easier to ground myself.

open-text response: Jkyg

He’s quite grounding actually, like I found when I was in therapy sessions and I’d be talking about stuff, it was quite difficult. I would kind of start to disassociate a little bit. I found that stroking him was actually really helpful for keeping myself there.

Usuv

##### Comforting and Self-Soothing

Young people established a relationship with Purrble, where they would seek comfort and companionship from it*:*

I felt like I had a friend and someone who cared for me.

open-text response: Rhhn

This was particularly useful for young people who expressed loneliness as a factor for their distress and self-harm:

I consider him sometimes like a puppy I always wanted to have so you know that the loyalty is there. You don’t need to worry about anything. You can share your stories to share your sadness with him.

Fcpy

Purrble is framed as a creature that needs to be cared for when it is distressed (indicated by small noises and rapid heartbeat); by caring for Purrble, young people were able to distract themselves from their own distress:

...having something outside of yourself to focus on and kind of almost take care of and it’s another set of quote unquote needs that you’re trying to focus on.

Fead

This “comforting” of an external presence meant that young people were better able to manage their emotions and for some that they could not engage with their self-harm:

I just love him so much. He’s actually my child and you can’t self-harm if you’re holding a fluffy little creature.

Usuv

...when Purrble is like anxious and his hearty rate is up I am so focused with trying to calm him down that I literally just, all my energy and focus goes on that, every anxiety I had and my head just goes. Because I am so concerned about the animal or Purrble that I forget, yes.

Fhac

From descriptions, it appeared that young people also used Purrble as a permission to prioritize their own well-being while they comforted Purrble, rather than just as a distraction:

It’s teaching self-soothing which is really important if you’re having self-harm urges because self-soothing is what you need in that situation rather than self-harm.

Lyco

I feel like people with anxiety, depression, self-harm thoughts don’t really think of themselves as something to be looked after, but having a little thing that gets anxious and stressed makes you want to look after things and by way of that you look after yourself more.

Usuv

##### Addressing Physical Manifestations of Distress

As Purrble is a tangible intervention, this allowed young people to address the physical symptoms of their emotional distress, such as their heart rate increasing when anxious:

I found that if I put it against my heart it felt, like, it was a cat or something. I found that helpful.

Bdbg

This participant described how they would hold Purrble to their chest to feel the repetitive heart vibration, which was used as a method to address anxiety symptoms, whereas other participants reported rhythmically stroking Purrble, fidgeting with the ears and feet to occupy their hands, or using the heartbeat to guide breathing exercises:

...if someone’s stressed out, having a bit of a panic, you might tell them to do some deep breaths and sometimes that can feel a bit ridiculous while you’re having a panic, but if you’re handed this little creature that’s also panicking, then you’re just going to naturally start to go, “It’s okay, sssh,” and just do deep breaths at the same time just to try and help him calm down and yourself calm down. So Purrble and deep breathing, very helpful.

Usuv

#### Stages of Change

##### Overview

Young people were clear about when they felt that Purrble would be useful. These views reflected when Purrble would be an effective, acceptable intervention, whereas at other points, Purrble would not be useful. This was frequently related to how able they felt they were to reduce their self-harm.

##### Stopping the Spiral

Purrble was discussed as a useful tool to prevent young people from spiraling into negative thought patterns and emotions:

When I was feeling sad, I think purrble was the most helpful and maybe where my anxiety was a bit lower, it was like starting to raise before it got to be like super anxious than purrble would be more helpful.

Ivjl

...everything just slowly build up when I meet those about things in life and purrble is somehow in this process of building it up as cutting it down bit by bit. So I don’t really reach the point where I just have very, very terrible feelings so yeah.

Fcpy

Across both populations, this was highly related to feelings of anxiety, which would lead to an overall lower mood or self-harm. However, the caveat of this was that Purrble was used when emotions were not too intense:

I think that purrble would come in useful as long as it’s [anxiety] not too intense.

Huiq

##### Breaking the Habit

In the interviews, the participants described their journey of self-harm. Young people often described where they felt they were on this journey and related Purrble use to their own stage. It was discussed that Purrble could be considered a useful intervention for those who felt they had lower engagement with self-harm:

I think yeah, for people who perhaps, who don’t self-harm as much, it’s not part of, it’s not become part of their routine maybe, I think it [purrble] would be a good at distracting them.

Agwn

It was discussed that Purrble could be considered a useful intervention for when someone felt they were recovering from habitual self-harm:

...definitely for anybody who is in recovery help from, you know what’s the word, it would help from relapsing.

Fhac

However, young people were mindful that self-harm is a difficult mindset to break:

...if it’s like people have done it for the last 10 years and it’s kind of part of their routine and it’s their only escape, it’s probably going to be quite hard to change that mindset.

Agwn

In this case, it was less likely that someone would easily shift to engaging with Purrble rather than self-harm. It was clear that there needed to be a motivation to change their self-harm behaviors before Purrble could be useful:

...anyone who is still quite deep in the self-harming might not want to come out because I remember when I was self-harming I didn’t even want to stop, I didn’t care to stop so any strategy that was told to me I kind of just ignore it. In my head I was like I am not hurting anyone else so why does anybody else care. So I wouldn’t care to stop and it’s kind of like a weird mentality.

Fhac

## Discussion

### Principal Findings

This study was the first to determine that Purrble, a socially assistive robot, acted as a feasible and acceptable intervention for young minority people at risk of self-harm by supporting ER in the moment of distress. Crucially, Purrble was found to interrupt the development of self-harmful thoughts and help prevent self-harmful behavior by promoting ER strategies. To the best of our knowledge, this is an important finding that has not been previously observed in this field.

Akin to Purrble, other digital interventions have been found to be feasible and acceptable among young people who self-harm [57-60]. However, these interventions are often much more complex than Purrble, requiring a high degree of engagement from participants and interpersonal relationships, for example, Reframe-IT, a web-based cognitive behavioral therapy program [57]; As Safe As Possible, 3-hour inpatient intervention supported by an app [58]; Crisis Care, an app to support coping for young people and parents [59]; and Village, a peer communication app [60]. In comparison, Purrble is an in situ device that allows participants to engage and disengage at their convenience without the need for external support. Therefore, this could be attractive to young people who are uncomfortable seeking help that includes an interpersonal element.

To date, these studies have provided limited evidence on the impact of digital interventions on mental health outcomes. Crisis Care and Village did not consider the impact on mental health outcomes in their trials [59,60]; Reframe-IT and As Safe As Possible found promising but nonsignificant effects [57,60]. Across interventions, including Purrble, better-powered trials are required to offer a clearer understanding of the intervention efficacy.

Between pilot studies, there was stronger evidence for the effective uptake of Purrble within LGBTQ+ participants than ethnic and minority youth, irrespective of their sexual orientation. This suggests that there could be something attractive about Purrble as an intervention mechanism for those who identify as LGBTQ+. This may relate to LGBTQ+ youth regularly engaging with strategies to self-manage their own mental health [61] owing to concerns of stigma or discrimination from health care staff [62,63]. Recognizing that LGBTQ+ young people are frequent users of technology [64-67], it is worth noting that Purrble, as a unique digital intervention designed to support self-regulation of emotions, may be particularly well-suited for this population. Further investigation as to whether and why Purrble is appealing to this group would offer a greater understanding of interventions for mental health among LGBTQ+ youth.

In pilot study 2, participants were less engaged with Purrble, which could be related to lower levels of need (lower anxiety and depressive symptoms and self-harm experiences at baseline). However, this may also have been linked to mental health interventions being less acceptable among racial and ethnic minority groups. Although mental health is relatively consistent between racial and ethnic groups [68], research suggests that some minority groups feel less need for mental health treatment [68] and that there are some barriers related to cultural differences [69,70], for example, reluctance to discuss mental health outside of family [71] or religious and spiritual beliefs that shape attitudes toward treatment [71-73]. In 1 interview, an ethnic minority participant spoke about stigma within the family home, how this had impacted her perception of mental health, and how she handled her own mental health. This suggests that there may have been underlying cultural or social differences that were not captured as part of the study.

### Limitations

Despite some novel and positive findings, these pilot studies had several limitations. First, they both included small samples of mainly cisgender female participants, a common pattern among self-harm intervention studies [31]. This restricts how widely we can generalize the results of each pilot study. Additional research is required to understand whether Purrble is more acceptable among cisgender female participants than among those who identify as male or gender diverse or whether these samples are reflective of self-harm research generally.

Second, although both LGBTQ+ and ethnic and racial minority youth are thought to be at risk of self-harm, we did not measure self-harm behavior in daily surveys, and participants did not have to have current experiences of self-harmful thoughts or behavior to take part. Therefore, these results may be less applicable to those who are highly engaged in self-harm. This is highlighted by our qualitative findings, which indicate that Purrble may not be as helpful for those who were struggling to break from the self-harm mindset.

Furthermore, both minority groups have diverse identities in the samples, which may influence how they perceive or use an intervention. This may be related to cultural or social differences between identities or between intersectional identities. In the future, a larger sample would be needed to explore these nuances to determine whether Purrble is better suited to some minority identities and why this might be the case.

Finally, ESM data were limited within this study, as participants were only asked to respond once a day. Although daily surveys are a strength to capture within-person fluctuations in participants’ lives, the results cannot provide clear temporal pathways of Purrble use in situ when dealing with distress. However, in this context, we support our preliminary findings using qualitative data to strengthen our understanding.

### Conclusions

These pilot studies were the first to demonstrate that Purrble appears to be a promising in situ intervention to support ER among young minority people at risk of self-harm. Young people reported Purrble as a feasible and acceptable tangible device for distress, supporting the downregulation of emotions to prevent some experiences of self-harm and emotional distress.

Overall, LGBTQ+ youth demonstrated a higher level of engagement with Purrble, and there was a greater perceived impact, in comparison with young people from ethnic and racial minority backgrounds. Despite this, ethnic and racial minority youth still described how Purrble was used to support them in the moment of distress, including self-harm. However, further research is necessary to determine the effectiveness of Purrble across larger samples and to what extent it is linked to long-term self-harm and ER.

## Appendix

Multimedia Appendix 1 Safeguarding procedures.

Multimedia Appendix 2 Interview topic guide.

Multimedia Appendix 3 Experience sampling measures.

Multimedia Appendix 4 Experience sampling measures.

## Abbreviations

ER: emotion regulation
ESM: experience sampling measure
GAD-7: Generalized Anxiety Disorder-7
LGBTQ+: lesbian, gay, bisexual, transgender, queer, and similar minority
PHQ-9: Patient Health Questionnaire-9
TWEETS: Twente Engagement with E-health Technologies Scale

## Data Availability

The data sets generated and analyzed during this study are available from the corresponding author upon reasonable request.
