# Supplementary material for: Investigating the Feasibility, Acceptability, and Appropriation of a Socially Assistive Robot Among Minority Youth at Risk of Self-Harm: Results of 2 Mixed Methods Pilot Studies
Source: JMIR Form Res. 2023 Nov 22;7:e52336. doi: 10.2196/52336 (PMC10701649; doi:10.2196/52336)
Supplement: Multimedia Appendix 1 [file formative_v7i1e52336_app1.docx]

# Safeguarding procedures

To ensure the safety of participants, several safeguarding measures were taken. These were explained to the participants prior to providing consent via the information sheet and during the study briefing. All participants received a letter addressed to their GP, which explained that they were taking part in an experience sampling study looking at mood across a 21-day period and may seek additional support. Included were the participants’ GAD-7 and PHQ-9 scores, so that GPs may have more information about why the young person was seeking support. No information was presented that this was a self-harm or minority youth study to avoid unwanted disclosure for the participant. While participants were encouraged to pass on this letter, they were not obligated to inform the research team of their decision.

As part of baseline review, participants responses to self-harmful thoughts (with and without suicidal intention) and behaviours items were checked. If participants had reported experiencing any of these during the previous 6 months, they were required to complete a safety plan (Stanley & Brown, 2012) as part of the study briefing. This was conducted with an expert researcher present to help or discuss any thoughts which occurred at this point. Participants were encouraged to use their personalised safety plan if they felt distressed during the study, but that they were free to withdraw if they wished.

During the 21-day ESM period, participants' responses were checked once a day. If participants reported thoughts of self-harm for the first time, a wellbeing check was conducted via phone call the following day. This included an assessment of immediate risk, encouraging help-seeking, and completing a safety plan (Stanley & Brown, 2012). This aligns with a similar ESM safeguarding procedure (Williams et al., 2022).

At the end of all ESM surveys, participants were provided with signposting information. This reminded them to seek support if they felt distress, citing services such as their GP and helplines (Samaritans, Papyrus, Kooth), alongside informal support; family and friends.

**References**

Stanley B, Brown GK. Safety planning intervention: a brief intervention to mitigate suicide risk. Cognitive and behavioral practice. 2012 May 1;19(2):256-64.

Williams AJ, Arcelus J, Townsend E, Michail M. Feasibility and acceptability of experience sampling among LGBTQ+ young people with self-harmful thoughts and behaviours. Frontiers in psychiatry. 2022 Aug 17;13:916164.
