# Supplementary material for: Investigating the Feasibility, Acceptability, and Appropriation of a Socially Assistive Robot Among Minority Youth at Risk of Self-Harm: Results of 2 Mixed Methods Pilot Studies
Source: JMIR Form Res. 2023 Nov 22;7:e52336. doi: 10.2196/52336 (PMC10701649; doi:10.2196/52336)
Supplement: Multimedia Appendix 2 [file formative_v7i1e52336_app2.docx]

# Post-interview: Topic guide

1. To start off, what were your overall opinions and thoughts about the study?

[Prompt: length of study, daily surveys, delivery of study]

[Prompt: study expectation vs having completed]

2. Is there anything you would have changed about the study?

3. *IF: The participant had a safety plan ->* Did you use the safety plan during this study? Did you find that it was helpful at all? Why?

4. Over the course of the study, how did you engage with Purrble? If you didn’t not engage with Purrble, why do you think this was?

[Prompt: what was engagement to you (e.g. picking Purrble up)? What situations might these be?]

[Prompt: was there something about Purrble you didn’t really like?]

5. Did you find Purrble useful?

6. Were there circumstances or difficulties which you think Purrble helped you with? What were these? How was Purrble helpful?

7. When you think about the stressful or emotionally overwhelming situations you had over the past month, were there any where Purrble was consistently helpful?

[Prompt: How and why do you think Purrble helped you in those situations?]

[Prompt: Compared to other strategies you may have used before having Purrble or alongside Purrble how was the effect of using Purrble similar or different to what you would normally do otherwise?]

8. Were there aspects of Purrble which you didn’t like or didn’t find helpful?

9. Generally, how useful do you think Purrble would be for other young people who may have difficulties with their emotions?

[Prompt: How might Purrble be effective or not-effective with young people dealing with self-harmful thoughts or behaviours?]

10. What do you think would be useful for young people who are struggling with self-harm?

11. Have you found certain strategies or treatments helpful for your experiences of self-harm?

12. Do you have any other thoughts that you’d like to share regarding the study?

[Prompt: how do you think we could improve it? What would you have done differently?]

Thank you so much for taking part. It’s been really useful to hear your thoughts and opinions on the study and thank you for taking part in the initial steps of this study.

Is there anything you would like to ask me before we finish?
