# Supplementary material for: Investigating the Feasibility, Acceptability, and Appropriation of a Socially Assistive Robot Among Minority Youth at Risk of Self-Harm: Results of 2 Mixed Methods Pilot Studies
Source: JMIR Form Res. 2023 Nov 22;7:e52336. doi: 10.2196/52336 (PMC10701649; doi:10.2196/52336)
Supplement: Multimedia Appendix 3 [file formative_v7i1e52336_app3.docx]

# ESM daily measures

From the SUPREME CORT study, the adapted version of GAD-7 and PHQ-9 were adopted [40]. These measures follow the same structure as the originals discussed at baseline assessment but ask about mood over the course of a day rather than two weeks. These were rated on a 4-point Likert scale ranging from 0 (not at all) to nearly every day (3). The total score of the adapted-GAD-7 offers insight into anxiety symptoms, while the total score of the adapted PHQ-7 indicates depressive symptoms across the day. Neither ESM measures are used as clinical indicators however.

Emotion regulation was assessed using one measure built of 4 items [41,42]. These measured the extent to which participants were able to engage in emotional regulation strategies; distraction, expressive suppression, social sharing, and reappraisal over the course of the day. Items were rated on a 7-point Liker scale from 1 (not at all) to 7 (a lot).

One binary item regarding thoughts of self-harm was taken from the SIGMA study [43]. On a 7-point Likert scale from 1 (not at all) to 7 (very much) participants were asked whether they had considered harming themselves that day.

A bespoke measure was included as an indicator of Purrble engagement; this had previously been used with highly anxious students [37 , 38]. The measure was excluded from week 1 of the surveys but included for each daily survey across weeks 2-3. Three items asked about Purrble engagement over the cause of the day (frequency, duration, perceived impact) using a 6-point Likert scale. Following this, participants were asked to rate whether Purrble had a positive or negative impact on how they felt that day and offered an option to explain via open-text response.

# ESM items

The listed ESM items are presented in the order that they were seen by participants.

**Emotion regulation measure[41-42]**

This first set of questions will ask you about emotional regulation. Please think about to what extent you have experienced these thoughts of feelings over the course of the day. (7-point Likert scale 1-7)

- To what extent did I try to distract myself from my feelings?

*<not at all> ← → <a lot>*

- To what extent did I try to suppress the expression of my feelings?

*<not at all> ← → <a lot>*

- To what extent did I talk about my feelings with others?

*<not at all> ← → <a lot>*

- To what extent did I try to look at the cause of my feelings from a different perspective?

*<not at all> ← → <a lot>*

**Mental health measures (SUPREME CORT[40])**

This next section asks about your mood over the course of the day.

**Over the day**, how often have you been bothered by the following problems? (4-point Likert scale 0-3)

- Feeling nervous, anxious or on edge

*<not at all> ← → <nearly all day>*

- Not being able to stop or control worrying

*<not at all> ← → <nearly all day>*

- Worrying too much about different things

*<not at all> ← → <nearly all day>*

- *Trouble relaxing*

*<not at all> ← → <nearly all day>*

- Being so restless that it is hard to sit still

*<not at all> ← → <nearly all day>*

- Becoming easily annoyed or irritable

*<not at all> ← → <nearly all day>*

- Feeling afraid as if something awful might happen

*<not at all> ← → <nearly all day>*

- Little interest or pleasure in doing things

*<not at all> ← → <nearly all day>*

- Feeling down, depressed, or hopeless

*<not at all> ← → <nearly all day>*

- Trouble falling or staying asleep, or sleeping too much

*<not at all> ← → <nearly all day>*

- Feeling tired or having little energy

*<not at all> ← → <nearly all day>*

- Poor appetite or overeating

*<not at all> ← → <nearly all day>*

- Feeling bad about yourself — or that you are a failure or have let yourself or your family down

*<not at all> ← → <nearly all day>*

- Trouble concentrating on things, such as reading the newspaper or watching television

*<not at all> ← → <nearly all day>*

- Moving or speaking so slowly that other people could have noticed? Or the opposite — being so fidgety or restless that you have been moving around a lot more than usual

*<not at all> ← → <nearly all day>*

- Thoughts that you would be better off dead or of hurting yourself in some way.

*<not at all> ← → <nearly all day>*

**Self-harm measure (SIGMA[43])**

The next questions will briefly ask about self-harm.

Have you considered harming yourself today? (7-point Likert scale 1-7)

*<not at all> ← → <very much>*

**Purrble engagement measure (Oxford Purrble study[37, 38])**

***(Questions only shown weeks 2-3)***

The final section will ask about how you may have used Purrble today (6-point sliding scale 0-5)

- How many times did you engage with Purrble today?

*<not even once (0)> ← → <we were inseparable (more than 5 times)>*

- Did it usually make any difference to how you felt at the time?

*<not at all> ← → <very much>*

**•** If yes, then positive / negative impact? [checkbox]

• Optional open-ended text to expand on the answer [textarea]
