# Supplementary material for: Investigating the Feasibility, Acceptability, and Appropriation of a Socially Assistive Robot Among Minority Youth at Risk of Self-Harm: Results of 2 Mixed Methods Pilot Studies
Source: JMIR Form Res. 2023 Nov 22;7:e52336. doi: 10.2196/52336 (PMC10701649; doi:10.2196/52336)
Supplement: Multimedia Appendix 4 [file formative_v7i1e52336_app4.docx]

# Additional table: Participant demographics by pilot sample

All demographic information was self-reported in open-text responses. We have collapsed mixed race into one category for this table, however, there was a high degree of diversity among those who identified as mixed race and whether they explicitly stated their races.

|  | **Pilot 1** | **Pilot 2** |
| --- | --- | --- |
| Age M (SD) | 21.4 (2.7) | 21.1 (2.8) |
| Ethnicity N (%) | | |
| *White British* | 18 (85.7) | - |
| *Black British* | 1 (4.8) | 2 (10.5) |
| *White minority* | - | 1 (5.2) |
| *Mixed race* | 1 (4.8) | 5 (26.3) |
| *Latino/a* | 1 (4.8) | - |
| *Asian Indian* | - | 3 (15.8) |
| *Asian Chinese* | - | 5 (26.3) |
| *Arabic* | - | 1 (5.2) |
| *Pakistani* | - | 2 (10.5) |
| Sexual orientation | | |
| *Heterosexual* | - | 10 (52.6) |
| *Asexual* | 1 (4.8) | 1 (5.2) |
| *Demisexual* | - | 1 (5.2) |
| *Bisexual* | 10 (47.6) | 4 (21.1) |
| *Gay/Lesbian* | 6 (28.6) | 2 (10.5) |
| *Pansexual* | 1 (4.8) | 1 (5.2) |
| *Queer* | 3 (14.3) | - |
| Gender identity | | |
| *Cisgender female* | 14 (66.7) | 16 (84.2) |
| *Cisgender male* | - | 1 (5.2) |
| *Non-binary* | 2 (9.5) | 2 (10.5) |
| *Agender* | 1 (4.8) | - |
| *Transgender man* | 2 (9.5) | - |
| *Transmasculine (demi-boy)* | 1 (4.8) | - |
| *Questioning* | 1 (4.8) | - |
| Employment | | |
| *Currently unemployed* | 1 (4.8) | 2 (10.5) |
| *Part-time employment* | 1 (4.8) | 1 (5.2) |
| *Full-time employment* | 4 (21.1) | 6 (31.8) |
| *School or college student* | 4 (21.1) | 3 (15.8) |
| *University student* | 10 (47.6) | 7 (36.8) |
| *Volunteering* | 1 (4.8) | - |
| Self-harm history in last 6 months (%) | | |
| *Self-harm thoughts* | 95.2 | 84.2 |
| *Suicidal thoughts* | 61.9 | 57.9 |
| *Self-harm behaviour* | 81.0 | 52.6 |
